# Extrinsic Calibration for a Modular 3D Scanning Quality Validation Platform with a 3D Checkerboard

**DOI:** 10.3390/s24051575

**Published:** 2024-02-29

**Authors:** Mirko Kaiser, Tobia Brusa, Martin Bertsch, Marco Wyss, Saša Ćuković, Gerrit Meixner, Volker M. Koch

**Affiliations:** 1 Biomedical Engineering Lab, Bern University of Applied Sciences, 2502 Biel, Switzerland; 2 Laboratory for Movement Biomechanics, ETH Zurich, 8092 Zürich, Switzerland; sasa.cukovic@hest.ethz.ch; 3 Usability and Interaction Technology Lab, Heilbronn University, 74081 Heilbronn, Germany; gerrit.meixner@hs-heilbronn.de

**Keywords:** 3D checkerboard, 3D scanning, extrinsic 3D calibration, point cloud registration, testing and validation platform

## Abstract

Optical 3D scanning applications are increasingly used in various medical fields. Setups involving multiple adjustable systems require repeated extrinsic calibration between patients. Existing calibration solutions are either not applicable to the medical field or require a time-consuming process with multiple captures and target poses. Here, we present an application with a 3D checkerboard (3Dcb) for extrinsic calibration with a single capture. The 3Dcb application can register captures with a reference to validate measurement quality. Furthermore, it can register captures from camera pairs for point-cloud stitching of static and dynamic scenes. Registering static captures from TIDA-00254 to its reference from a Photoneo MotionCam-3D resulted in an error (root mean square error ± standard deviation) of 0.02 mm ± 2.9 mm. Registering a pair of Photoneo MotionCam-3D cameras for dynamic captures resulted in an error of 2.2 mm ± 1.4 mm. These results show that our 3Dcb implementation provides registration for static and dynamic captures that is sufficiently accurate for clinical use. The implementation is also robust and can be used with cameras with comparatively low accuracy. In addition, we provide an extended overview of extrinsic calibration approaches and the application’s code for completeness and service to fellow researchers.

## 1. Introduction

Optical 3D scanning is widely used in medical applications [1,2,3,4,5,6,7,8,9], and scanning systems that track patient movement in 3D are becoming ubiquitous. Some of these can capture the human upper body, in particular the human back, in various postures and movements such as standing upright, bending forward, and bending sideways. There are two options for capturing the human back during these postures and movements: Follow the patient with a single capturing system or use multiple 3D scanning systems. Using a single capturing system requires a person or actuator to follow the patient’s movement. In addition, the movement of the system must also be tracked, which requires user intervention, is a complex task, and can result in a loss of accuracy [10,11,12,13,14]. Conversely, the use of multiple 3D scanning systems allows the patient’s movement to be captured with minimal user intervention, but this approach requires repeated extrinsic calibration between these systems, which can be time consuming.

Many authors have proposed solutions for extrinsic calibration. However, most extrinsic calibration methods are specific to LiDAR systems used in autonomous driving for long-range measurements. Other methods are specific to LiDAR and color (RGB) mono camera or color mono and depth camera, require texture, require a camera with high resolution, require a time-consuming calibration process involving multicapture, and do not come with implementation details or publicly available code (Table A1 in Appendix A).

Beltran et al. [15] published a toolbox for automatic calibration of sensor pairs consisting of LiDAR and mono and stereo camera devices in any possible combination. Their calibration target contains four round holes and four ArUco markers. Yan et al. [16] published a calibration toolbox for intrinsic and extrinsic LiDAR-camera calibration for autonomous driving vehicles. Their toolbox contains a rich set of sensor calibration methods, including inertial measurement units (IMUs) and radar. Their calibration board contains four round holes and a 2D checkerboard pattern. Domhof et al. [17] published an extrinsic calibration tool to calibrate sensor setups consisting of LiDAR, camera, and radar sensors with a calibration board with four round holes.

Zhang et al. [18] proposed a two-step method for extrinsic calibration between a sparse 3D LiDAR and a thermal camera. The method involved two steps: Extrinsic calibration between LiDAR and a visual camera, followed by extrinsic calibration between the visual camera and the thermal camera. Their 3D checkerboard was derived from work by Rangel et al. [19] and Skala et al. [20].

None of these solutions is applicable to all scanning methodologies, provides sufficient calibration accuracy with a single capture, includes implementation details, and has publicly available code. In this paper, we present a simple, robust, and practical 3D checkerboard, including an algorithm and software, to calibrate different 3D systems with each other with a single capture. The work by Beltran et al., Yan et al., Domhof et al., Zhang et al., Rangel et al., and Skala et al. shows the potential of extrinsic calibration using a calibration target with holes and inspired our 3D checkerboard approach, which can be used to calibrate diverse methodologies such as structured light (SL) [21,22], active stereo (AS) [22,23], and time of flight (ToF) [24]. The validation results for the systems used here are evaluated and compared to classical approaches in the literature.

## 2. Materials and Methods

**Figure 1:**
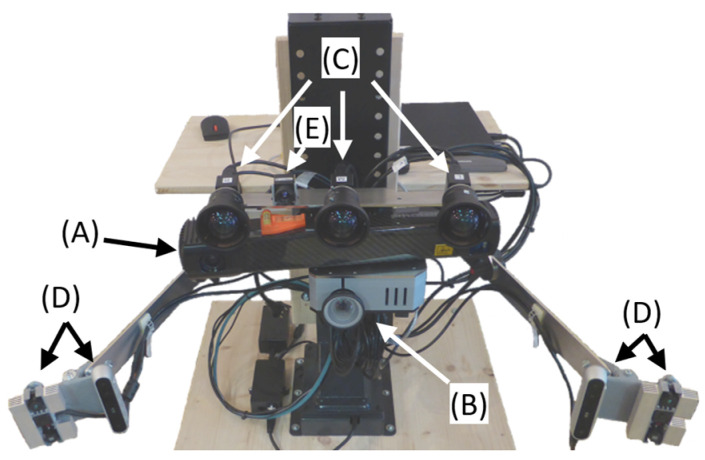
Modular quality validation platform (DMQV) for 3D scanning with (A) Photoneo MotionCam-3D M+ (MotionCam 1), (B) DLP LightCrafter 4500, (C) 2D HIKROBOT cameras, (D) Orbbec Astra Mini and Intel D415, and (E) InfiRay Micro III thermal camera.

The Biomedical Engineering Lab has built a modular quality-validation platform (DMQV, Figure 1) for 3D scanning consisting of various 3D scanners, 3D cameras, and 3D scanning methodologies. A Photoneo (Photoneo s.r.o, Bratislava, Slovakia) MotionCam-3D camera (MotionCam 1; Figure 1A) [21] is used as a reference for static and dynamic captures because it has an accuracy of <0.3 mm. The platform also contains a DLP LightCrafter 4500 pattern projector from Texas Instruments (Texas Instruments, Dallas, Texas, USA; Figure 1B) [25] and three monochrome 2D cameras from HIKROBOT (HIKROBOT, Hangzhou, Zhejiang, China; MV-CA023-10UM; Figure 1C) [26]. The DLP LightCrafter 4500 and 2D cameras from HIKROBOT combined with TIDA-00254, a structured light machine vision application from Texas Instruments [22], can capture high-quality 3D images from static scenes. In combination with BoofCV (Peter Abeles, version 0.41) [23], the system can capture 3D images from dynamic scenes with stereo and trinocular vision. The platform also includes two consumer-grade 3D cameras, the Orbbec (Orbbec, Shenzhen Shi, China) Astra Mini and the Intel (Intel, Santa Clara, California, USA) D415 (Figure 1D), which use single-shot structured light and active stereo, respectively.

The DMQV has been developed to investigate the minimum key parameters required to capture the human back shape [27], build models that allow spinal alignment to be estimated from back shape, and investigate correlations between back shape and spinal alignment.

**Figure 2:**
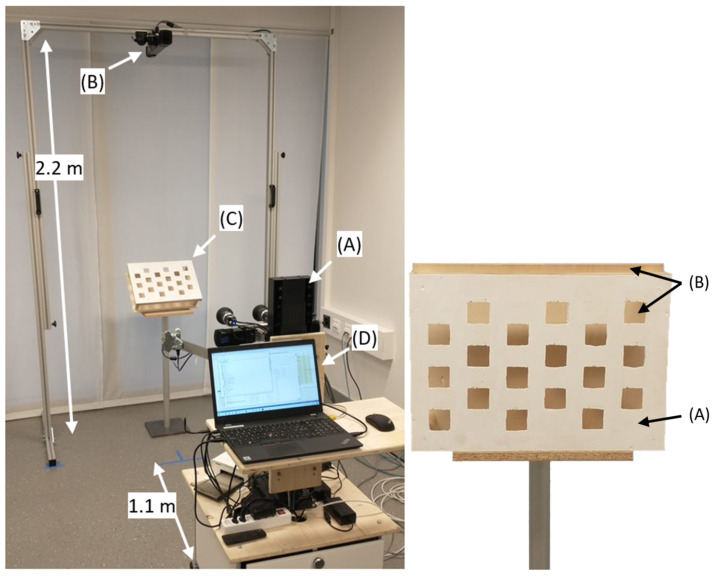
(**Left**): Extended DMQV platform with (A) DMQV platform, (B) additional MotionCam-3D M+ from above (MotionCam 2), (C) 3D checkerboard in 45°, and (D) laptop with software. (**Right**): 3D checkerboard with (A) a plane with holes in the front and (B) a solid plane behind.

The Functional Spinal Biomechanics group at ETH used the DMQV platform at the Spiraldynamik MedCenter Zurich and an extended version at the Balgrist University Hospital in Zurich to capture the human back in various postures and movements. The extended platform (Figure 2, left) includes an additional Photoneo MotionCam-3D (MotionCam 2) to capture the patient from above during static and dynamic forward bending. The distance between the patient’s back and MotionCam 1, DLP LightCrafter, and HIKROBOT cameras was 1.1 m, the distance between the patient’s back and the Orbbec Astra Mini and Intel D415 was 0.9 m, and the distance between MotionCam 2 and the floor was 2.2 m.

The DMQV platform was enhanced with a 3D checkerboard (Figure 2, right) to extrinsically calibrate the systems and to register the captures from the systems with each other. The 3Dcb consists of a plane with holes (Figure 2, right, A) arranged in six distinct rows and columns, giving 18 holes in total. Our 3D checkerboard was 21 cm × 29.7 cm (A4) in size, and each hole was 2 cm × 2 cm. The sizes of the checkerboard and holes can be scaled according to the intended application, the distance between the camera and the checkerboard, and the quality of the systems in use. Low-quality systems require a larger checkerboard and larger holes. Situated 10 cm behind the plane with holes is another completely solid plane (Figure 2, right, B). Some systems, such as the Intel D415, tend to smooth over the holes when the pattern projected by the inbuilt projector is not visible in the stereo camera pair; the second plane reflects the pattern, and thus the holes are detected more robustly.

**Figure 3:**
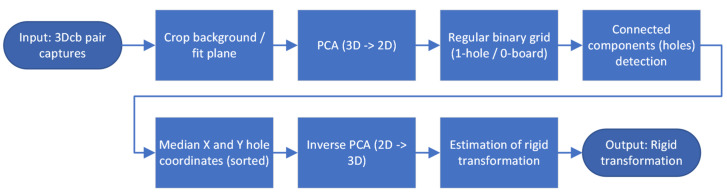
Flowchart for calculating the rigid transformation between two 3D scanning systems from a pair of 3D checkerboard captures.

**Figure 4:**
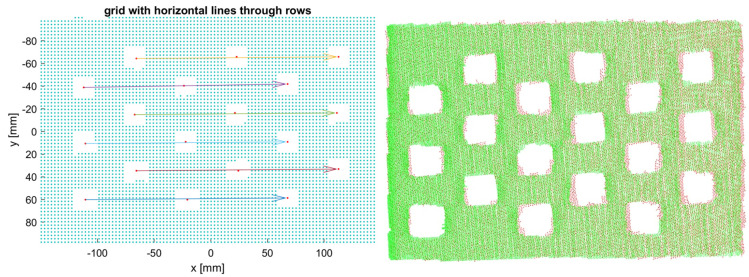
(**Left**): Binary 2D projection (blue points) of a 3Dcb capture with sorted (colored arrows) hole medians (red points). (**Right**): Registered 3D checkerboards from two Photoneo MotionCam-3Ds (green and red) after applying the estimated geometric transformation.

The 3D captures of the 3Dcb from each camera are evaluated in pairs (Algorithm 1, Figure 3): First, the background is cropped by using a rough estimate of the distance between the 3Dcb and the camera. Then a plane is fitted (pcfitplane in MATLAB; MathWorks, Natick, Massachusetts, USA, version R2021b), which keeps only the points from the plane of the 3Dcb with the holes; all points outside of the fitted plane are removed, including the points from the solid plane behind the holes. A principal component analysis (pca in MATLAB) is then performed to align the larger dimension of the 3Dcb to the X-axis and project it into 2D. Next, the projection of the 3Dcb is rasterized with a regular grid, i.e., with a resolution of 3 mm, and labeled with 1 for hole, no nearest neighbor (findNearestNeighbors in MATLAB) found within the resolution, and 0 for board. This binary image is then checked for connected components (bwconncomp in MATLAB), which detects all holes. The median (median in MATLAB) is then calculated for both X and Y coordinates for all components. The median points of all holes are sorted (sort in MATLAB) in X and Y directions (Figure 4, left) to detect the arrangement and orientation of the 3Dcb. The medians are then transformed back into the 3D space using the inverse PCA transform. By exploiting the known arrangement and orientation of the median points, a rigid transformation is estimated (estimateGeometricTransform3D in MATLAB) between pairwise system captures. The estimated rigid transformation can then be applied directly to the captures from each system to transform the point cloud into the coordinate system of all other systems (Figure 4, right).
**Algorithm 1.** MATLAB pseudocode to calculate the rigid transformation between two 3D scanning systems from a pair of 3D checkerboard captures.Pc2   %point cloud from capture of 3Dcb from system 2 pc1_ref %point cloud from system 1 (reference coordinate system) function Checkerboards3d_estimateT(pc2, pc1_ref)  % remove background pc2_noBackground = abs(pc2-distEstimation) <= eps pc1_noBackground = abs(pc1_ref-distEstimation) <= eps for pc = pc2_noBackground, pc1_noBackground do   % fit plane, do PCA to transform from 3D into 2D  planeModel = pcfitplane(pc)  [pcaPlane,coeff,mu] = pca(planeModel)   % create regular binary grid  for [x,y] = min(pcaPlane):resolution:max(pcaPlane)   point_nn = findNearestNeighbors(pcaPlane, [x,y], 1)   if abs(point_nn-[x,y])>resolution    zGrid(x,y) = 1   end if  end for   % detect connected components (holes)  CC = bwconncomp(zGrid)   % calculate median coordinates of holes, sort them and   % use inverse PCA to transform back into 3D  Median_cc = median(CC)  holeMedians = sort(Median_cc)  holeMedians3D = holeMedians * transpose(coeff) + mu end for  % estimate the geometric transformation between hole medians from both checkerboards tFormEst = estimateGeometricTransform3D(holeMedians3D_2, holeMedians3D_1,’rigid’) return tFormEst end function

**Figure 5:**
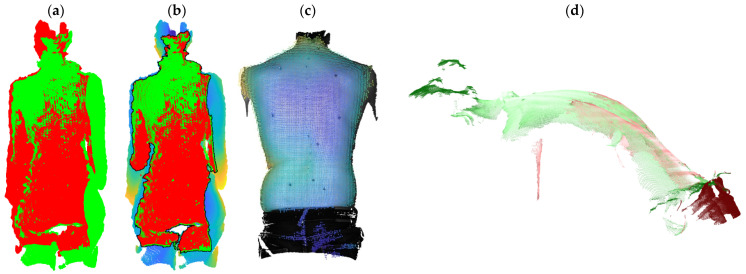
Use Case 1: (**a**) Example of registration of left (red) and right (green) Orbbec Astra Mini camera pair. (**b**) Example of the same registration (colored, blue) with overlapping region (black border) of left (red) and right (green) camera pair. Use Case 2: (**c**) Example of registration of TIDA-00254 (colored) to its reference from Photoneo MotionCam-3D (textured). Use Case 3: (**d**) Example of registration of the human back surface captured from above (green) and behind (red) with the Photoneo MotionCam-3D.

The DMQV platform, including the 3Dcb, was used to capture static standing upright and static and dynamic bending forward and sideways at the Spiraldynamik Med Center Zurich on 72 patients (mean age 54 ± 16 years). The patients attended the medical center with a diverse spectrum of spinal disorders, including back pain, limited range of mobility, or simply for a routine checkup. The extended platform version was used at the Balgrist University Hospital in Zurich on 22 patients who attended with idiopathic scoliosis (mean age 18 ± 4 years). The height of the DMQV platform was adjusted for each patient to optimize the field of view. Therefore, captures of the 3Dcb in the vertical position (0°) and at 45° were taken from all systems after each patient. These 3Dcb captures were used to register the systems to each other with all 94 patients. Three use cases were evaluated (Table 1): Use Case 1 registers captures from left and right camera pairs for static standing upright (Figure 5a), Use Case 2 registers static standing upright captures to its reference capture (Figure 5c), and Use Case 3 registers captures from above and behind for dynamic forward bending (Figure 5d).

The Photoneo MotionCam-3D uses structured light [21]; therefore, the captures from MotionCam 1 and MotionCam 2 for dynamic forward bending (Use Case 3) must be made sequentially. To reduce the delay between the captures, a hardware trigger was used to daisy-chain the two cameras. An iterative closest point (ICP) optimization was performed for the overlapping region after the 3Dcb registration to correct for the remaining delay between the captures.

The metrics used to assess the quality of registration are the root mean square error (RMSE) and standard deviation (SD). The RMSE and SD are calculated using the nearest neighbors of all points for the overlapping region between the captures from the different systems. The overlapping region was defined as follows: a point $p_{1}$ from system 1 overlaps if there is at least one point $p_{2}$ from system 2 within a specified radius ρ, i.e., 6 mm, around the surface normal $n_{1}$ at that point: $\left|n_{1}\times \left(p_{2}-p_{1}\right)\right|<\rho$, where $p_{2}-p_{1}$ gives a vector pointing from $p_{2}$ to $p_{1}$, the cross product with $n_{1}$ gives a vector that is orthogonal to $n_{1}$ (definition of the cross product), and taking the norm of this resulting vector gives the distance between point $p_{2}$ and $p_{1}$ orthogonal to the normal vector $n_{1}$ at point $p_{1}$.

## 3. Results

Use Case 1: Register captures from left and right camera pairs for static standing upright.

**Figure 6:**
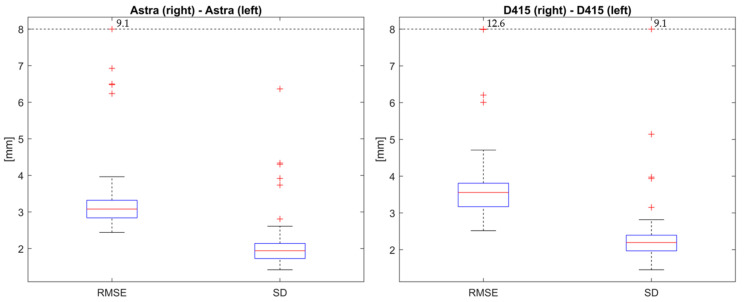
RMSE and SD for overlapping region at a distance of 0.9 m after registration for left and right camera pair for standing upright (Use Case 1). (**Left image**): Orbbec Astra Mini; (**Right image**): Intel D415.

The median RMSE for the overlapping region at a distance of 0.9 m for the left and right Orbbec Astra Mini camera pair (Figure 5b) after registration was 3.1 mm (Figure 6, left). The median SD was 1.9 mm. The median RMSE for the overlapping region at a distance of 0.9 m for the left and right Intel D415 camera pair after registration was 3.6 mm (Figure 6, right). The median SD was 2.2 mm.

Use Case 2: Register static standing upright captures to its reference capture.

**Figure 7:**
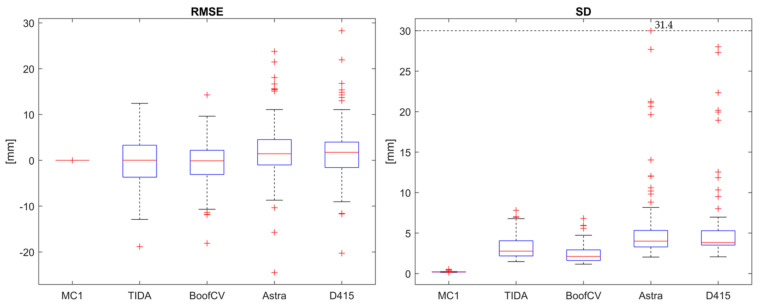
RMSE (**left image**) and SD (**right image**) for the overlapping region at distances between 0.9 m and 1.1 m after registration against its reference capture from MotionCam 1 for standing upright (Use Case 2); For MotionCam 1 (MC 1), TIDA-00254, BoofCV, Orbbec Astra Mini and Intel D415.

The median RMSE and SD for the overlapping region at distances between 0.9 m and 1.1 m for the captures from all systems and the MotionCam 1 as reference (Figure 5c) after registration (Figure 7) are shown in Table 2.

Use Case 3: Register captures from above and behind for dynamic forward bending.

**Figure 8:**
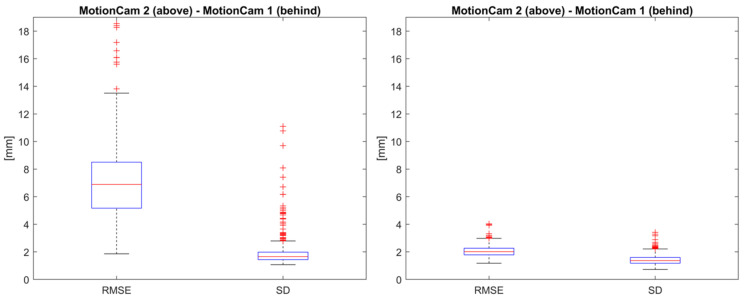
RMSE and SD for overlapping regions at a distance of 1.1 m after registration of above (MotionCam 2) and behind (MotionCam 1) camera pair for dynamic forward bending (Use Case 3). (**Left image**): without ICP; (**Right image**): with ICP.

The median RMSE for the overlapping regions at a distance of 1.1 m (Figure 5d) for the camera pair above (MotionCam 2) and behind (MotionCam 1) after registration was 6.9 mm (Figure 8, left). The median SD was 1.7 mm. An additional ICP registration for the overlapping region after the 3Dcb registration to correct for the delay between the captures from cameras 1 and 2 resulted in a median RMSE for the overlapping regions of 2.0 mm and a median SD of 1.4 mm (Figure 8, right).

## 4. Discussion

The median RMSE of 0 mm and the median SD of 0.2 mm for the overlapping region for the captures from the MotionCam 1 with itself as reference (Use Case 2) show that the 3D checkerboard leads to an estimation of a geometric transformation that is sufficiently accurate for clinical use. The higher RMSE and SD values of TIDA-00254, Orbbec Astra Mini, and Intel D415 reflect the accuracy of these systems. The RMSE and SD values are similar to the accuracy values stated by the manufacturers (Table 3), which is to be expected. The Intel D415 in particular tends to smooth out holes, but this is mitigated by the solid plane behind.

Furthermore, interference between these systems required that the captures be made sequentially, within a few seconds, and therefore involuntary movements such as swaying and breathing are included in the error values. Moreover, the voluntary movement between captures increases the error values, especially for dynamic bending (Use Case 3). The ICP registration reduced the median error value to 2.0 mm. Furthermore, the angle between MotionCam 2 and MotionCam 1 was 90°, so the 3Dcb was captured at 45°, and the overlapping area of the human back was also largely captured by both cameras with an incidence angle of 45°.

The literature on the registration of 3D captures and considerations for practical applications mostly focuses on camera–LiDAR calibration, covers only parts of our proposed solution, and mostly presents only results. Beltran et al. [15] used their calibration toolbox with a 1.4 m wide calibration target with four round holes. The resulting mean error for monocular–LiDAR calibration using 30 frames of three calibration target poses was 8.2 mm. Unfortunately, they do not state error values for their real test environment for stereo–stereo calibration. Our practical application showed error values between 0 mm and 3.6 mm for a single target pose with a single frame. We only used distances of around 1.1 m, whereas they used distances up to 6 m. The question arises: Which calibration target and algorithm perform better for which systems and target distances. Since our application is limited to distances around 1.1 m, future work could investigate the performance of our proposed solution at larger distances.

Yan et al. [16] used their calibration toolbox with a 1.2 m wide calibration board with four round holes and a 2D checkerboard pattern. Their toolbox contains a rich set of various sensor calibration methods but is specific to autonomous driving, such as camera–LiDAR calibration. Furthermore, they do not state error values for real test environments.

Domhof et al. [17] used their extrinsic calibration tool with a 1.5 m wide calibration board with four round holes. The resulting mean error for stereo–LiDAR calibration using 29 target locations within approximately 5 m was 15 mm. Stereo–stereo calibration was not investigated.

Zhang et al. [18] used their method for extrinsic calibration with a 3D checkerboard approximately 56 cm wide and consisting of 44 round holes. Low-cost cameras such as the Intel D415 and Orbbec Astra would have failed to detect all holes with a single capture. Furthermore, Zhang et al. suggest collecting more than 40 image pairs with the checkerboard in various positions, angles, and distances. Stereo–stereo calibration was not investigated, no error values were reported, and no implementation details or public code were provided.

The work by Beltran et al. [15], Yan et al. [16], Domhof et al. [17], Zhang et al. [18], Rangel et al. [19], and Skala et al. [20] demonstrates the potential of extrinsic calibration using a calibration target with holes. Unfortunately, none of their work was directly applicable to our specific setup, but it inspired our 3D checkerboard approach. Repeated calibration was necessary because the platform was adjusted to each patient, and the low-cost systems required a very robust method. Therefore, we implemented a robust approach requiring only a single capture. The work by Zhang et al. [18], Rangel et al. [19], Skala et al. [20], and our own research show that our 3D checkerboard and registration approach is applicable to further modalities, such as time of flight, and can even be extended to thermal cameras (Figure 1). This will be the focus of future work.

The holes of the proposed 3Dcb are arranged in a regular pattern on a single plane. The question arises whether a unique but irregular cubic arrangement could improve the estimated 3D transformation. The holes of the proposed 3Dcb are square. We investigated whether round holes would lead to better results; this was not the case, but the shape of the holes could be further investigated.

We also used multiple 3Dcb captures with different positions and locations of the checkerboard, but we did not find significant improvement over a single capture. The comparison between multiple captures and single capture could be further investigated and quantified.

## 5. Conclusions

The Biomedical Engineering Lab has built a modular quality validation platform for 3D scanning (DMQV) consisting of various 3D scanners, 3D cameras, and 3D scanning methodologies, including a 3D checkerboard for extrinsic calibration. The 3D checkerboard extrinsic calibration can be used to register 3D captures between different systems, to fuse these captures, and to compare various quality validation parameters between all systems. The registration of 3D captures with the 3Dcb requires only a single additional capture. The registration approach does not require texture and is therefore applicable to further modalities such as time of flight. The DMQV has been tested and validated with two studies for the systems detailed in Table 3.

The results show that our 3Dcb implementation provides accurate registration and is robust, simple, fast to use, and generalizable. The median RMSE and median SD of the overlapping regions after the registration of the 3D captures from SL with TIDA (0.02 mm ± 2.9 mm), Astra Mini (1.5 mm ± 4.0 mm), and Intel D415 (1.7 mm ± 3.9 mm) deviate only a few millimeters from the reference capture with the Photoneo MotionCam-3D and reflect the accuracy values provided by the manufacturers (Table 3). In addition, the 3Dcb registrations for the fusion of static captures from low-cost camera pairs and dynamic captures from a Photoneo MotionCam-3D camera pair were achieved with comparable errors.

We provide a complete pipeline for the registration of any 3D scanning methodology, including the CAD model of the 3Dcb and publicly available code (see Supplementary Materials) to facilitate further research on this topic. We believe that the integration of our extrinsic 3D calibration pipline will facilitate the use of setups involving multiple adjustable 3D scanning systems and thereby promote their dissemination in clinical research and practical applications.

## Figures and Tables

**Table 1:** Use cases evaluated in this paper.

Use Case	Posture–Movement	Systems
1: Register captures from left and right camera pairs	Static standing upright	2× Orbbec Astra, 2× Intel D415
2: Register captures to its reference capture	Static standing upright	TIDA-00254, BoofCV, 2× Orbbec Astra, 2× Intel D415, Photoneo MotionCam-3D
3: Register captures from above and behind	Dynamic forward bending	2× Photoneo MotionCam-3D

**Table 2:** Median RMSE (median SD) for the overlapping region at distances between 0.9 m and 1.1 m for all systems against its reference.

MotionCam 1	TIDA-00254	BoofCV	2× Orbbec Astra Mini	2× Intel D415
0 mm (0.2 mm)	0.02 mm (2.9 mm)	0.1 mm (2.1 mm)	1.5 mm (4.0 mm)	1.7 mm (3.9 mm)

**Table 3:** Systems evaluated in this paper with methodology (structured light—SL; active stereo—AS) used, and resolution and accuracy stated by the manufacturer.

System	Methodology	Resolution	Accuracy
Photoneo MotionCam-3D M+	SL	1680 × 1200 and 1120 × 800	error <0.3 mm at 0.9 m
TIDA-00254	SL	912 × 1140 and 1920 × 1200	error ~1 mm at 1 m
BoofCV	AS	912 × 1140 and 1920 × 1200	error ~1 mm at 1 m
Intel D415	AS	1280 × 720	error <2% up to 2 m
Orbbec Astra Mini	SL	640 × 480	error <3 mm at 1 m

## Data Availability

The data presented in this study are openly available in ETH Research Collection at https://doi.org/10.3929/ethz-b-000640083.

## Supplementary Materials

The MATLAB code presented in this study is openly available on GitHub at https://github.com/mkaisereth/Extrinsic3DCalibration, (accessed on 28 February 2024).

## Appendix A

**Table A1:** Literature with extrinsic calibration (see Table A2 and Table A3 for an extended overview).

Author & Reference	Sensor Type	Checkerboard	Multicapture or Single Capture	Texture Required	Code Available
J. Beltran [15]	LiDAR–camera (stereo, mono)	Calibration target with four round holes andArUco markers	Multicapture	No	Velo2cam
G. Yan [16]	LiDAR–camera (mono)	Calibration target with four round holes andcheckerboard pattern	Multicapture	Yes	OpenCalib
J. Domhof [17]	Radar–camera (stereo)–LiDAR	Calibration target with four round holes	Multicapture	No	Multi_sensor_ calibration
J. Zhang [18]	LiDAR–camera–thermal	2D checkerboards and 3D checkerboard	Multicapture	Yes	No
J. Rangel [19]	Thermal–RGB-D camera	3D checkerboard	Multicapture	Yes	No
K. Skala [20]	Thermal–RGB-D camera	3D checkerboard	Multicapture	Yes	No
**Proposed**	**Depth cameras (Structured light–active stereo–ToF)**	**3D checkerboard**	**Single capture**	**No**	**Extrinsic3D** **Calibration**

**Table A2:** Extended overview of literature with extrinsic calibration with publicly available code. Columns are author and reference, sensor type, name of toolbox and operating system–platform.

Author and Reference	Sensor Type	Toolbox Name	Operating System–Platform
C. Guindel [28]	LiDAR–stereo	Velo2cam	ROS
J. Beltran [15]	LiDAR–camera (stereo, mono)	Velo2cam	ROS
R. Unnikrishnan [29]	Camera–LiDAR	LCCT	MATLAB
A. Geiger [30]	LiDAR–ToF–Camera (stereo)	LIBCBDETECT	MATLAB
G. Yan [31]	LiDAR–camera (mono)	OpenCalib	C++
G. Yan [16]	LiDAR–camera (mono)	OpenCalib	C++
J. Domhof [17]	Radar–camera (stereo)–LiDAR	Multi_sensor_calibration	ROS
J. K. Huang [32]	LiDAR–camera (mono)	extrinsic_lidar_camera_calibration	MATLAB
A. Dhall [33]	LiDAR–camera (mono, stereo)	lidar_camera_calibration	ROS
M. Velas [34]	LiDAR–RGB camera (mono)	but_calibration_camera_velodyne	ROS
L. Yin [35]	LiDAR–camera (mono)	multimodal_data_studio	MATLAB
P. C. Su [36]	RGB-D cameras	RGBD_CameraNetwork_Calibration	C++
**Proposed** **(Kaiser et al.)**	**Depth cameras (Structured light–active stereo–ToF)**	**Extrinsic3DCalibration**	**MATLAB**

**Table A3:** Extended overview of literature with extrinsic calibration with author and reference, sensor type, checkerboard type or scene, features used for correspondence, whether the approach requires multiple captures or a single capture, publication type, whether the approach requires texture and whether the approach is specific for autonomous driving. Legend: Multicapture (MC), single capture (SC), multitarget (MT), journal (J), conference (C), online (O), no explicit information (X), and not applicable (n.a.).

Author and Reference	Sensor Type	Checkerboard or Scene	Features	Multicapture or Single Capture	Publication Type	Texture Required	Autonomous Driving
M. Lindner [37]	PMD ToF–RGB camera (mono)	2D checkerboard	Plane	MC	C	Yes	X
S. Fuchs [38]	ToF	2D checkerboard	Plane (dark–bright)	MC	C	Yes	X
J. Zhu [39]	ToF–passive stereo	2D checkerboard	Plane (dark–bright)	MC	C	Yes	X
H. Zou [40]	Laser profilers	Spheres	Spheres	MC	J	No	No
J. Schmidt [41]	ToF	Scene	Point correspondence	MC	C	Intensity	X
H. Lee [42]	LiDAR	Planar objects from Scene	Plane correspondence	MC	J	No	Yes
S. Chen [43]	LiDAR	Spheres	Sphere center and corresponding points	MC	J	No	Yes
C. Guindel [28]	LiDAR–stereo	Calibration target with four round holes	Plane, Circles and point correspondence	MC	C	No	Yes
J. Beltran [15]	LiDAR–camera (stereo–mono)	Calibration target with four round holes and ArUco markers	Plane, Circles, point correspondence and ArUco markers	MC	J	No–Yes	Yes
Y. M. Kim [44]	ToF–camera (stereo)	2D checkerboard	Corners	MC	C	Yes	No
D. Scaramuzza [45]	LiDAR–camera (mono)	Scene	Natural features	MC	C	Yes	Yes
R. Unnikrishnan [29]	Camera–LiDAR	2D checkerboard	Corners and Plane	MC	O	Yes	Yes
Q. Zhang [46]	Camera–LiDAR	2D checkerboard	Plane	MC	C	Yes	Yes
A. Geiger [30]	LiDAR–ToF–Camera (stereo)	Multiple 2D checkerboards	Corners and Planes	SC (Multitarget)	C	Yes	X
L. Zhou [47]	Stereo–LiDAR	2D checkerboard	Plane and Line correspondences	MC	C	Yes	Yes
Q. Wang [48]	LiDAR–camera	3x 2D checkerboard	Planes and Corners	MC	J	Yes	Yes
S. Verma [49]	Camera (mono)–LiDAR	2D checkerboard	Planes and Corners	MC	C	Yes	Yes
J. Ou [50]	LiDAR–camera (mono, stereo)	2D checkerboard	Corners, intensity and Plane	MC	J	Yes	Yes
X. Gong [51]	LiDAR–camera	Trihedron	Planes	MC	J	Yes	Yes
G. Yan [31]	LiDAR–camera (mono)	Calibration target with four round holes and checkerboard pattern	Circles and Corners	MC	O	Yes	Yes
Y. An [52]	LiDAR–camera (mono)	2D checkerboard	Plane and Corners	MC	J	Yes	Yes
S. A. Rodriguez F. [53]	LiDAR–camera (mono)	Circle	Circle	MC	C	Yes	Yes
G. Yan [16]	LiDAR–camera (mono)	Calibration target with four round holes and checkerboard pattern	Circles and Corners	MC	J	Yes	Yes
J. Zhang [18]	LiDAR–camera–thermal	2D checkerboards and 3D checkerboard	Corners and circles	MC	C	Yes	Yes
J. Domhof [17]	Radar–camera (stereo)–LiDAR	Calibration target with four round holes	Circles	MC	C	No	Yes
E. S. Kim [54]	LiDAR–camera (mono)	2D checkerboard	Corners and Plane	MC	J	Yes	Yes
Y. Wang [55]	LiDAR–camera (mono)	Review	n.a.	n.a.	C	n.a.	Yes
A. Khurana [56]	LiDAR–camera (mono, stereo)	Review	n.a.	n.a.	J	n.a.	Yes
J. Nie [57]	LiDAR–camera (mono)	Review	n.a.	n.a.	C	n.a.	Yes
D. J. Yeong [58]	LiDAR–camera (mono, stereo)	Review	n.a.	n.a.	J	n.a.	Yes
P. An [59]	LiDAR–camera (mono)	2D checkerboards	Corners and Plane	Multitarget	J	Yes	Yes
J. Persic [60]	LiDAR–Radar	Triangular trihedralcorner reflector	Triangle and Plane	MC	C	No	Yes
J. K. Huang [32]	LiDAR–camera (mono)	Planar square targets	Plane and Corners	MC	J	Yes	Yes
A. Dhall [33]	LiDAR–camera (mono, stereo)	Planar boards with ArUco tags	Corners and Edges	MC	O	Yes	Yes
M. Velas [34]	LiDAR–RGB camera (mono)	Calibration target with four round holes	Circles and edges	SC	C	Yes	Yes
L. Yin [35]	LiDAR–camera (mono)	2D checkerboard	Corners and plane	MC	J	Yes	Yes
H. Liu [61]	RGB-D cameras	Spheres	Sphere center	MC	J	Yes	X
A. Perez-Yus [62]	RGB camera–Depth camera	Line observations	Lines	MC	J	Yes	Yes
C. Daniel Herrera [63]	RGB camera–Depth camera	2D checkerboard	Corners and Plane	MC	J	Yes	No
J. Chaochuan [64]	RGB-D cameras	Calibration tower	Circles	MC	J	Yes	No
Y. C. Kwon [65]	RGB-D cameras	Circles and spheres	Circles	MC	J	Yes	No
Z. Wu [66]	RGB camera–Depth camera	3D Checkerboard	Corners	MC	C	Yes	No
R. Avetisyan [67]	RGB-D cameras	2D Checkerboard and Markers	Corners and Markers	MC	C	Yes	No
R. S. Pahwa [68]	PMD depth camera (ToF)	2D checkerboard	Corners and Plane	MC	C	Yes	No
D. S. Ly [69]	Mono cameras	Scene	Lines	MC	J	Yes	No
W. Li [70]	3D scanner–optical tracker	3D benchmark	Point set (ICP)	MC	J	No	No
M. Ruan [71]	Depth cameras	Spherical target	Shere center	MC	C	No	No
N. Eichler [72]	Depth cameras	human motion	Skeletal joints	MC	J	No	No
B. S. Park [73]	RGB-D cameras	3D Charuco board	QR code and feature points	MC	J	Yes	No
J. Guan [74]	Mono cameras	Spheres	Sphere center	MC	J	Yes	No
P. C. Su [36]	RGB-D cameras	Spheres	Sphere center	MC	J	Yes	No
J. Rangel [19]	Thermal–RGB-D camera	3D checkerboard	Circular holes	MC	C	Yes	No
K. Skala [20]	Thermal–RGB-D camera	3D checkerboard	Rectangular holes	MC	J	Yes	No
**Proposed (Kaiser et al.)**	**Depth cameras (Structured light–active stereo–ToF)**	**3D checkerboard**	**Rectangular holes and Plane**	**SC**	**J**	**No**	**No**
